# Discovery of KIRREL as a biomarker for prognostic stratification of patients with thin melanoma

**DOI:** 10.1186/s40364-018-0153-8

**Published:** 2019-01-14

**Authors:** Sebastian Lundgren, Helena Fagerström-Vahman, Cheng Zhang, Liv Ben-Dror, Adil Mardinoglu, Mathias Uhlen, Björn Nodin, Karin Jirström

**Affiliations:** 1 Department of Clinical Sciences Lund, Oncology and Pathology, Lund University, Lund, Sweden; 2 Science for Life Laboratory, Department of Proteomics, School of Biotechnology, Royal Institute of Technology, Stockholm, Sweden

**Keywords:** Melanoma, KIRREL, NEPH1, Prognosis

## Abstract

There is a great unmet clinical need to identify patients with thin primary cutaneous melanomas (T1, Breslow thickness ≤ 1 mm) who have a high risk for tumour recurrence and death from melanoma. Kin of IRRE-like protein 1 (KIRREL/NEPH1) is expressed in podocytes and involved in glomerular filtration. Screening in the Human Protein Atlas portal revealed a particularly high expression of KIRREL in melanoma, both at the mRNA and protein levels. In this study, we followed up on these findings and examined the prognostic value of KIRREL in a population-based cohort.

Immunohistochemical expression of KIRREL was examined in tissue microarrays with a subset of primary tumours and paired lymph node metastases from an original cohort of 268 incident cases of melanoma in the Malmö Diet and Cancer study. KIRREL mRNA expression was examined in 103 melanoma cases in The Cancer Genome Atlas (TCGA).

Membranous/cytoplasmic expression of KIRREL was detected in 158/185 (85.4%) primary tumours and 18/19 (94.7%) metastases. High expression of KIRREL was significantly associated with several unfavourable clinicopathological factors.

High KIRREL protein expression was an independent factor of reduced recurrence free and melanoma specific survival, particularly in thin melanomas, even outperforming absolute thickness and ulceration (HR = 30.85; 95% CI 1.54–616.36 and HR = 6.32 95% CI 1.19–33.65). High mRNA levels of KIRREL were not significantly associated with survival in TCGA.

In conclusion, KIRREL is not only a novel potential diagnostic marker for melanoma, but may also be a useful prognostic biomarker for improved stratification of patients with thin melanoma. These findings may be of high clinical relevance and therefore merit further validation.

**Electronic supplementary material:**

The online version of this article (10.1186/s40364-018-0153-8) contains supplementary material, which is available to authorized users.

Cutaneous melanoma is the most aggressive and deadliest form of skin cancer with a rising incidence globally [1]. Kin of IRRE protein (KIRREL) is a member of a podocin binding protein family, which primary physiological role is in the renal glomeruli where it safeguards selective ultrafiltration [2]. As of yet, research regarding the expression and role of KIRREL in cancer has been sparse. A recent study demonstrated that high expression of KIRREL is associated with unfavorable clinicopathological factors and poor prognosis in gastric cancer [3], and KIRREL has also been implicated in the carcinogenesis of pancreatic cancer [4]. We conducted a screening of mRNA and protein levels of KIRREL across several cancers and normal tissues in the Human Protein Atlas (HPA) portal (www.proteinatlas.org) [5], which revealed a particularly high expression in melanoma. The aim of this study was therefore to further investigate the expression, clinicopathological correlates and prognostic impact of KIRREL in melanoma, both at the protein and mRNA levels.

## Results and discussion

**Fig. 1:**
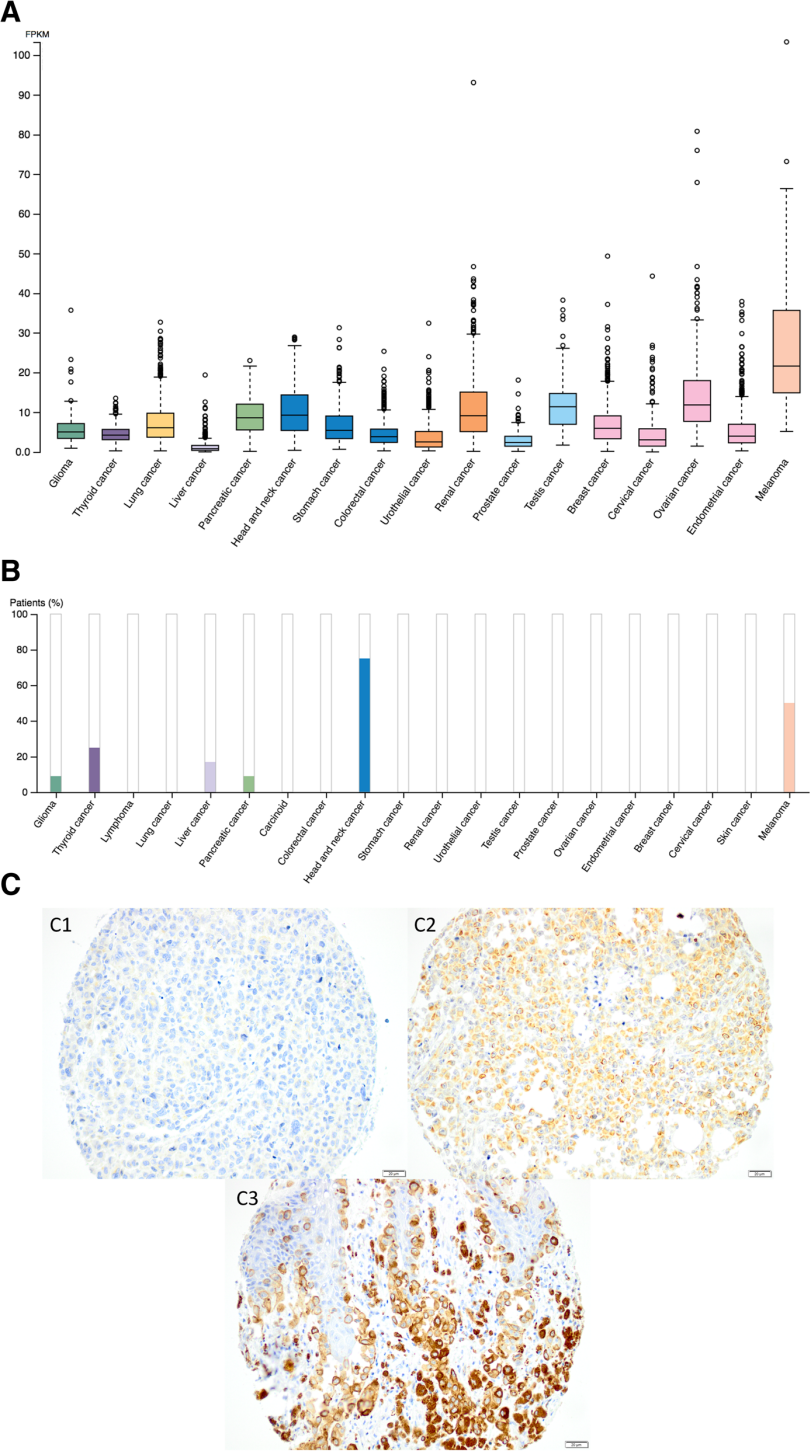
Overview of KIRREL expression at the mRNA and protein levels in different cancer types and sample images of immunohistochemical staining in melanoma. **a** KIRREL mRNA expression overview and (**b**) KIRREL protein expression summary across several cancer types. **c** Immunohistochemical images from melanoma cases in the Malmö Diet and Cancer study, exemplifying tumors with 1) negative, 2) intermediate, and 3) high expression

mRNA and protein expression of KIRREL in various types of cancers in the HPA are shown in Fig. 1 a, b. KIRREL expression was further examined by immunohistochemical analysis of 226 cases of incident melanoma in the population-based cohort Malmö Diet and Cancer study, from which tissue microarrays had been constructed as previously described (Additional file 1) [6].

Sample immunohistochemical images of KIRREL staining are shown in Fig. 1c. Digital images of KIRREL expression in 12 cases of melanoma are also shown in the HPA: https://www.proteinatlas.org/ENSG00000183853-KIRREL/pathology/tissue/melanoma.

Membranous and cytoplasmic KIRREL expression was detected in 158/185 (85.4%) of evaluable primary tumours and in 18/19 (94.7%) of evaluable metastases. Pairwise comparisons between mean expression of KIRREL in primary tumours and metastases revealed a higher expression in the latter, however this difference was not significant (*p* = 0.17).

**Fig. 2:**
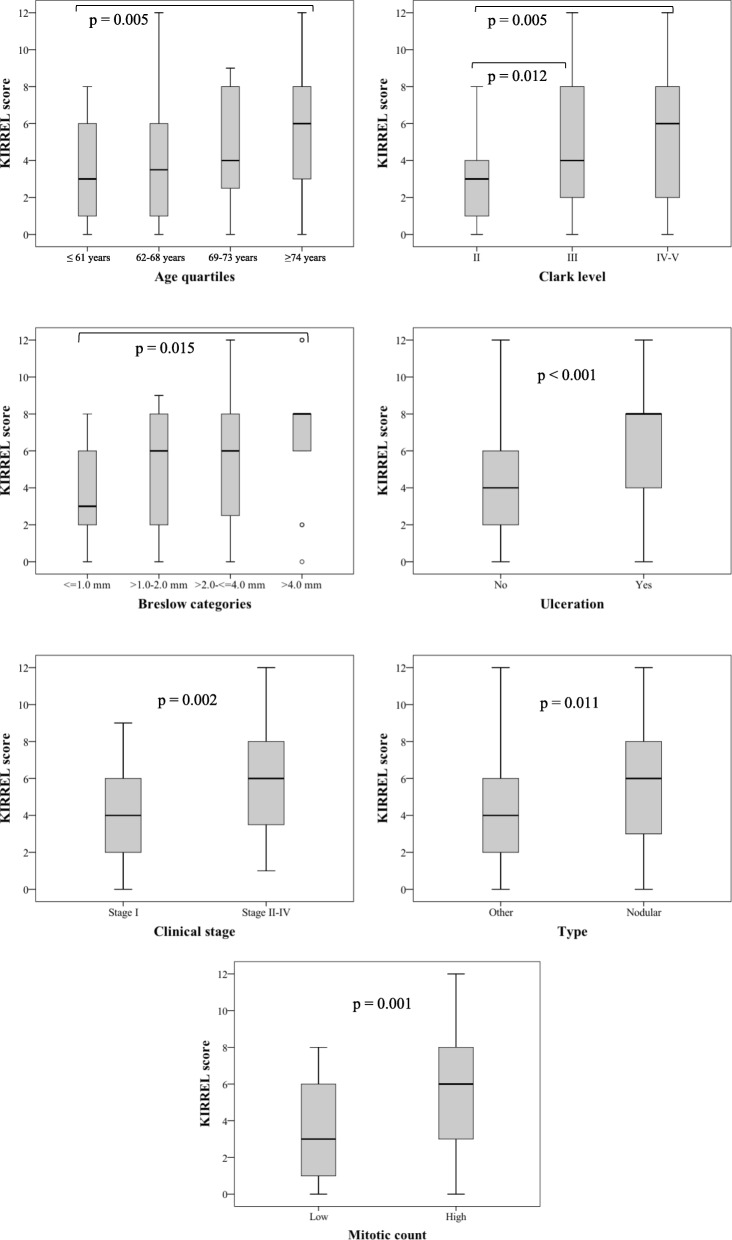
Distribution of KIRREL expression in relation to clinicopathological factors. Box plots visualizing the distribution of the full KIRREL score in relation to clinicopathological factors. Only significant *p*-values are shown. Error bars indicate 95% confidence interval

The associations of KIRREL expression with clinicopathological factors are detailed in Additional file 2, and significant associations are visualized in Fig. 2. High expression of KIRREL was significantly associated with several unfavourable clinicopathological traits, including high Clark level and Breslow stage, ulceration, clinical stage, and high mitotic count. These findings are in line with a recent study on gastric cancer that demonstrated that KIRREL expression is associated with several adverse clinicopathological factors [3, 7].

**Fig. 3:**
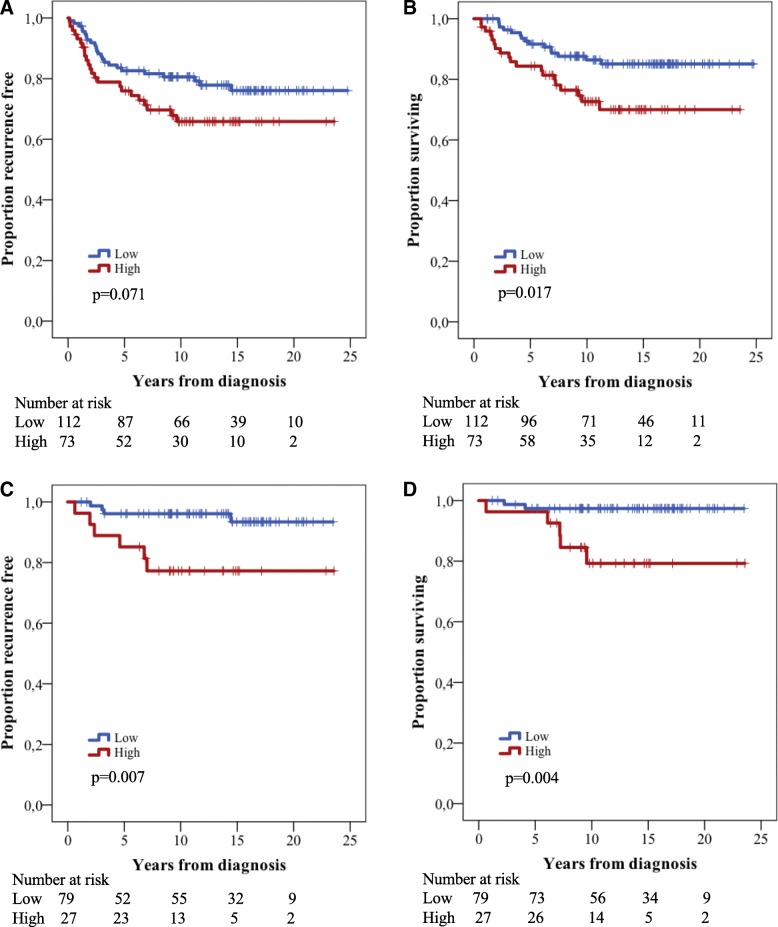
Kaplan-Meier estimates illustrating differences in clinical outcome stratified by KIRREL expression. **a** Recurrence free survival in the entire cohort. **b** Melanoma specific survival in the entire cohort. **c** Recurrence free survival in thin melanomas only. **d** Melanoma specific survival in thin melanomas only

Next we examined the impact of immunohistochemical KIRREL expression on patient outcome. After a median follow-up of 11.8 years (range 0.6–24.8), 113 (50%) of patients had died, 36 (31.8%) of whom from melanoma, and recurrent disease was denoted in 50 (22.1%) cases. As shown in Fig. 3, high KIRREL expression was a significantly associated with reduced melanoma-specific survival (MSS) (*p* = 0.017), and a trend, however non-significant, towards a shorter recurrence free survival (RFS) (*p* = 0.071). These associations were confirmed in univariable Cox regression analysis (Hazard ratio [HR] = 1.69 95% Confidence interval [CI] 0.95–2.99 and HR = 2.23 95% CI 1.13–4.00, respectively), but did not remain significant in multivariable analysis (data not shown).

In thin melanomas (Breslow thickness ≤ 1 mm) high protein expression of KIRREL was significantly associated with both poor RFS and reduced MSS (*p* = 0.004 and *p* = 0.007, respectively) (Fig. 3). As shown in Additional file 3, these associations were confirmed in univariable Cox regression analysis and remained significant in multivariable analysis (HR = 30.8; 95% CI 1.34–616, HR = 6.32 95% CI1.19–33.65 respectively), even outperforming well-established prognostic factors such as absolute thickness in millimetre and presence of ulceration.

Thin melanomas already make up 70% of all newly diagnosed melanomas [8], a number that may increase due to efforts for earlier diagnosis, and although they can collectively be regarded as having a good prognosis, some tumours will metastasize and lead to death. Current clinical protocols still lack reliable biomarkers for stratification of patients with thin melanoma having high or low risk for recurrence, and the findings in this paper indicate that KIRREL might valuable in this regard.

In order to validate the findings at the protein level, data on mRNA expression of KIRREL was retrieved from 103 melanoma cases in TCGA. As only two cases had matched samples from the primary tumour and metastasis, no meaningful comparison could be performed. There was no significant association between KIRREL expression and clinical stage (data not shown) and information on other clinicopathological factors was not available.

KIRREL expression was not prognostic at the mRNA level (Additional file 4), and as only 3 cases were stage I, no subgroup analysis could be carried out for thin melanoma.

## Conclusions

This is the first study to describe the expression of KIRREL at the protein and mRNA levels in cutaneous melanoma. The results demonstrate a particularly high expression of KIRREL in melanoma compared to other types of solid cancer, in both primary tumours and metastases, hence highlighting its potential as a novel diagnostic adjunct. Moreover, KIRREL may also prove to be a useful biomarker for improved prognostic stratification of thin melanoma, an entity for which there is great unmet need for improved risk stratification. Further research into the role of KIRREL in the pathogenesis and progression of melanoma, and validatory studies regarding its clinical impact, are warranted.

## Additional files

Additional file 1: Materials and Methods. (DOCX 19 kb)

Additional file 2: Associations of KIRREL protein expression with clinicopathological factors in the entire cohort. (DOCX 22 kb)

Additional file 3: Cox regression analysis of hazard ratios for A) recurrence and B) death from melanoma in thin melanoma. (DOCX 23 kb)

Additional file 4: Kaplan-Meier curve illustrating overall survival according to high and low mRNA expression (median cutoff) in TCGA dataset. (PDF 45 kb)

## Abbreviations

CI: Confidence interval
FPKM: Fragments Per Kilobase Million
HPA: Human Protein Atlas
HR: Hazard ratio
KIRREL: Kin of IRRE like
MSS: Melanoma specific survival
RFS: Recurrence free survival
TCGA: The Cancer Genome Atlas
