# Supplementary material for: Discovery of KIRREL as a biomarker for prognostic stratification of patients with thin melanoma
Source: Biomark Res. 2019 Jan 14;7:1. doi: 10.1186/s40364-018-0153-8 (PMC6332842; doi:10.1186/s40364-018-0153-8)
Supplement: Supplementary file 1 — Materials and Methods. (DOCX 19 kb) [file 40364_2018_153_MOESM1_ESM.docx]

**Additional file 1: Material and methods**

**Study cohort**

Up until December 31^st^ 2008, 268 incident melanoma cases were identified in the prospective, population-based cohort Malmö Diet and Cancer Study, through the Swedish Cancer Registry and the Southern Sweden Regional Tumour Registry. Information on recurrence (local, regional or distant) was updated until 31 Dec 2017 from patient records and pathology reports. Information on vital status and cause of death was updated from the Swedish Cause of Death Registry and the Swedish Civil registry up until 31 Dec 2017. All cases with available archival tumour tissue underwent histopathological re-evaluation. Data on tumour location and Breslow-Clark staging were obtained from patient charts. Follow-up started at date of diagnosis and ended at recurrent disease, death, emigration or December 31^st^ 2017.

**Tissue microarray construction**

Paraffin embedded archival tumour tissues were collected if available, and in a total number of 226 (85.6%) cases, primary tumours were suitable for tissue microarray (TMA) construction . Viable and representative tumour areas were marked and from these areas three 0.6 millimetre cores were obtained from each tumour and subsequently mounted in a recipient block using a semiautomated arraying device (TMArrayer, Pathology Devices, Westminster, MD, USA). Paired metastases were collected in 31 cases and underwent the same procedure for tissue microarray construction. Data on mRNA levels and clinical outcome were retrieved from 103 cases of melanoma in The Cancer Genome Atlas.

**Immunohistochemical staining and data collection**

4 μm TMA-sections were automatically pre-treated with the PT-link system (DAKO, Glostrup, Denmark) and stained in a Autostainer Plus (DAKO, Glostrup, Denmark) with a polyclonal antibody; HPA 030458, Atlas Antibodies, Bromma, Sweden, diluted 1:100. The immunohistochemical staining was evaluated by two pathologists (HFV and KJ), who were blinded to clinical outcome. KIRREL was expressed in the cytoplasm and cell membrane and the fraction of positive cells was denoted as well as the intensity of staining (high, low, absent). A combined score ranging from 0-12 was then constructed by multiplying the fraction and intensity. For the survival analysis, low expression was defined as a combined score of 0-5 and high expression as 6-12, based on visual inspection of the Kaplan-Meier curves for all scores in relation recurrence free survival (RFS) and melanoma specific survival (MSS).

**Statistical analysis**

Non-parametric tests were used to illustrate differences in KIRREL expression (combined score) and clinicopathological factors. Wilcoxon rank sum test was applied to evaluate differences in KIRREL expression between primary tumours and metastases. Kaplan-Meier and log-rank test were applied to illustrate differences in RFS and MSS. Cox regression proportional hazard modelling was used to examine the impact of KIRREL expression on RFS and MSS. Only factors that were significant in the unadjusted analysis were included in the adjusted analysis. The significance level was set at p = 0.05 and all tests were two-sided. All statistical analyses were performed using SPSS 24.0 (Chicago, IL, USA).
