# Supplementary material for: Discovery of KIRREL as a biomarker for prognostic stratification of patients with thin melanoma
Source: Biomark Res. 2019 Jan 14;7:1. doi: 10.1186/s40364-018-0153-8 (PMC6332842; doi:10.1186/s40364-018-0153-8)
Supplement: Supplementary file 2 — Associations of KIRREL protein expression with clinicopathological factors in the entire cohort. (DOCX 22 kb) [file 40364_2018_153_MOESM2_ESM.docx]

**Additional file 2.** Associations of immunohistochemical KIRREL expression with clinicopathological factors in the entire cohort

|  | **Entire cohort** | | | **Thin (<=1 mm) melanoma** | | |
| --- | --- | --- | --- | --- | --- | --- |
| Factor | *N* | KIRREL score* | *P** | *N* | KIRREL score* | *P** |
| **Age** |  |  |  |  |  |  |
| Q1 (≤ 61 years) | 44 | 3.7, 3.0 (3.0) | 0.018 | 24 | 3.2, 3.0 (2.6) | 0.089 |
| Q2 (62-68 years) | 56 | 3.9, 3.5 (3.2) |  | 31 | 3.0, 3.0 (2.5) |  |
| Q3 (69-73 years) | 44 | 4.4, 4.0 (2.9) |  | 34 | 4.0, 3.5 (2.7) |  |
| Q4 (≥74 years) | 44 | 5.7, 6.0 (3.3) |  | 17 | 4.6, 4.0 (2.3) |  |
| **Sex** |  |  |  |  |  |  |
| Female | 93 | 4.4, 4.0 (3.3) | 0.958 | 54 | 3.6, 4.0 (2.6) | 0.870 |
| Male | 92 | 4.4, 4.0 (3.1) |  | 52 | 3.6, 3.0 (2.7) |  |
| **Clark level** |  |  |  |  |  |  |
| II | 58 | 3.3, 3.0 (2.4) | 0.008 | 57 | 3.2, 3.0 (2.4) | 0.323 |
| III | 80 | 4.6, 4.0 (3.1) |  | 45 | 4.1, 4.0 (2.8) |  |
| IV-V | 45 | 5.5, 6.0 (3.7) |  | 4 | 4.0, 3.0 (2.8) |  |
| **Breslow** |  |  |  |  |  |  |
| <=1mm | 106 | 3.6, 3.0 (2.6) | 0.004 |  | - |  |
| 1.1-<=2.0 | 32 | 4.8, 6.0 (3.1) |  |  | - |  |
| >2.0-<=4.0 | 32 | 5.2, 6.0 (3.6) |  |  | - |  |
| >4.0 | 13 | 7.1, 8.0 (3.9) |  |  | - |  |
| **Ulceration** |  |  |  |  |  |  |
| No | 152 | 4.0, 4.0 (3.0) | <0.001 | 102 | 3.6, 3.0 (2.6) | 0.249 |
| Yes | 32 | 6.3, 8.0 (3.3) |  | 4 | 5.2, 6.0 (3.4) |  |
| **Lymphocytic infiltration** |  |  |  |  |  |  |
| 0-1 | 57 | 5.0, 4.0 (3.0) | 0.059 | 29 | 4.4, 4.0 (2.5) | 0.050 |
| 2-3 | 127 | 4.1, 4.0 (3.2) |  | 77 | 3.3, 3.0 (2.6) |  |
| **Vascular invasion** |  |  |  |  |  |  |
| No | 173 | 4.4, 4.0 (3.2) | 0.883 | 104 | 3.6, 3.0 (2.6) | 0.216 |
| Yes | 12 | 4.4, 4.0 (3.2) |  | 2 | 6.0, 6.0 (2.8) |  |
| **Clinical stage** |  |  |  |  |  |  |
| I | 125 | 3.8, 4.0 (2.7) | 0.002 |  | - |  |
| II-IV | 24 | 6.1, 6.0 (3.1) |  |  | - |  |
| **Location** |  |  |  |  |  |  |
| Dorsal trunk | 42 | 4.0, 4.0 (3.0) | 0.613 | 32 | 3.4, 3.5 (2.5) | 0.776 |
| Frontal trunk | 24 | 3.9, 4.0 (3.1) |  | 14 | 3.6, 3.5 (3.0) |  |
| Upper extremities | 38 | 4.8, 4.0 (3.4) |  | 18 | 3.4, 3.0 (2.6) |  |
| Lower extremities | 51 | 4.5, 4.0 (3.2) |  | 27 | 3.8, 3.0 (2.7) |  |
| Head and neck | 27 | 5.1, 4.0 (3.1) |  | 14 | 4.4, 4.0 (2.5) |  |
| **Type** |  |  |  |  |  |  |
| SSM, LMM, Other | 136 | 4.0, 4.0 (3.0) | 0.011 | 103 | 3.5, 3.0 (2.6) | 0.055 |
| NMM | 48 | 5.4, 6.0 (3.3) |  | 3 | 6.7, 8.0 (2.3) |  |
| **Mitotic count** |  |  |  |  |  |  |
| <1/mm^2^ | 84 | 3.5, 3.0 (2.7) | 0.001 | 77 | 3.4, 3.0 (2.6) | 0.136 |
| >=1/mm^2^ | 101 | 5.1, 6.0 (3.4) |  | 29 | 4.1, 4.0 (2.6) |  |
| **Ki67 positivity** |  |  |  |  |  |  |
| 0-1% | 12 | 3.6, 2.5 (3.4) | 0.586 | 7 | 3.3, 2.0 (3.5) | 0.226 |
| 2-25% | 116 | 4.4, 4.0 (3.0) |  | 73 | 3.8, 4.0 (2.5) |  |
| >25% | 42 | 4.6, 3.0 (3.5) |  | 13 | 2.8, 2.0 (2.6) |  |

*Mean, median (SD)

SSM = Superficial spreading melanoma, NMM = Nodular malignant melanoma, LMM = Lentigo malignant melanoma
