# Supplementary material for: Discovery of KIRREL as a biomarker for prognostic stratification of patients with thin melanoma
Source: Biomark Res. 2019 Jan 14;7:1. doi: 10.1186/s40364-018-0153-8 (PMC6332842; doi:10.1186/s40364-018-0153-8)
Supplement: Supplementary file 3 — Cox regression analysis of hazard ratios for A) recurrence and B) death from melanoma in thin melanoma. (DOCX 23 kb) [file 40364_2018_153_MOESM3_ESM.docx]

**Additional file 3a. Cox regression analyses of hazard ratios for recurrence in patients with thin melanoma**

|  | **Univariable** | | |  | **Multivariable** | | |  |
| --- | --- | --- | --- | --- | --- | --- | --- | --- |
| **Factor** | N | HR | 95% CI | *P* | N | HR | 95% CI | *P* |
| **Age** | 106* | 0.97 | 0.89-1.04 | *0.378* |  |  |  |  |
|  | 139° | 0.97 | 0.91-1.03 | *0.349* |  |  |  |  |
| **Sex** | 52 vs 54* | 1.12 | 0.32-3.89 | *0.853* |  |  |  |  |
| Male vs Female | 67 vs 72° | 1.17 | 0.38-3.63 | *0.789* |  |  |  |  |
| **Clark level** | 77 vs 62 | 1.84 | 0.52-6.53 | *0.338* |  |  |  |  |
| II vs III-V | 42 vs 41° | 1.55 | 0.44-5.49 | *0.499* |  |  |  |  |
| **Breslow (mm)** | 106* | 26.52 | 1.94-363.53 | ***0.014*** | 93 | 23.34 | 0.66-825.87 | *0.083* |
|  | 139° | 24.70 | 2.22-274.36 | ***0.009*** |  |  |  |  |
| **Ulceration** | 4 vs 102* | 5.82 | 0.72-46.95 | *0.098* |  |  |  |  |
| Yes vs no | 4 vs 135° | 6.19 | 0.79-48.65 | *0.083* |  |  |  |  |
| **Lymphocytic infiltration** | 77 vs 29* | 0.51 | 0.14-1.83 | *0.303* |  |  |  |  |
| 2-3 vs 0-1 | 105 vs 34° | 0.60 | 0.18-1.99 | *0.404* |  |  |  |  |
| **Vascular invasion** | 2 vs 104* | 6.01 | 0.75-48.06 | *0.091* |  |  |  |  |
| Yes vs no | 3 vs 136° | 3.94 | 0.51-30.54 | *0.190* |  |  |  |  |
| **Type** | 3 vs 103* | 15.02 | 3.09-73.00 | ***0.001*** | 3 vs 90 | 7.87 | 0.79-78.61 | *0.079* |
| NMM vs SSM, LMM, Other | 4 vs 135° | 9.64 | 2.10-44.18 | ***0.004*** |  |  |  |  |
| **Mitotic count** | 29 vs 77* | 2.81 | 0.81-9.72 | *0.102* |  |  |  |  |
| >=1/mm^2 vs^ <1/mm^2^ | 34 vs 105° | 2.34 | 0.74-7.37 | *0.147* |  |  |  |  |
| **Ki67 positivity** | 13 vs 80* | 6.58 | 1.64-26.47 | ***0.008*** | 13 vs 80 | 52.84 | 2.68-1040.35 | ***0.009*** |
| >25% vs <=25% | 15 vs 107° | 5.07 | 1.43-18.06 | ***0.012*** |  |  |  |  |
| **KIRREL high vs low** | 27 vs 79 | 4.88 | 1.37-17.38 | ***0.014*** | 22 vs 71 | 30.85 | 1.54-616.36 | ***0.025*** |

*Only cases with evaluable KIRREL expression included in the analysis.

° Entire cohort of thin melanoma, including cases with evaluable KIRREL expression and cases which non-evaluable KIRREL expression.

Only variables with a p-value <=0.05 in the univariable analysis, in either the KIRREL-evaluated or in the extended cohort, were included in the multivariable analysis.

SSM = Superficial spreading melanoma, NMM = Nodular malignant melanoma, LMM = Lentigo malignant melanoma.

**Additional file 3b. Cox regression analyses of hazard ratios for death from melanoma in patients with thin melanoma**

|  | **Univariable** | | |  | **Multivariable** | | |  |
| --- | --- | --- | --- | --- | --- | --- | --- | --- |
| Factor | N | HR | 95% CI | *P* | N | HR | 95% CI | *P* |
| **Age (continuous)** | 106* | 0.99 | 0.90-1.09 | *0.811* |  |  |  |  |
|  | 139° | 1.00 | 0.92-1.08 | *0.933* |  |  |  |  |
| **Sex** | 52 vs 54* | 0.67 | 0.15-2.99 | *0.600* |  |  |  |  |
| Male vs Female | 67 vs 72° | 0.52 | 0.12-2.17 | *0.368* |  |  |  |  |
| **Clark level** | 42 vs 41* | 0.76 | 0.70-3.38 | *0.713* |  |  |  |  |
| II vs III-IV | 77 vs 62° | 0.74 | 0.18-3.09 | *0.677* |  |  |  |  |
| **Breslow (continuous)** | 106* | 8.21 | 0.42-161.25 | *0.166* |  |  |  |  |
|  | 139° | 9.67 | 0.54-172.30 | *0.122* |  |  |  |  |
| **Ulceration** | 4 vs 102* | 8.83 | 1.04-75.30 | ***0.046*** | 4 vs 102 | 5.97 | 0.66-53.69 | *0.111* |
| Yes vs no | 4 vs 135° | 10.66 | 1.28-88.84 | ***0.029*** |  |  |  |  |
| **Lymphocytic infiltration** | 77 vs 29* | 0.47 | 0.10-2.10 | *0.321* |  |  |  |  |
| 2-3 vs 0-1 | 105 vs 34° | 0.51 | 0.12-2.13 | *0.509* |  |  |  |  |
| **Vascular invasion** | 2 vs 104* | 8.43 | 1.01-70.25 | ***0.049*** | 2 vs 104 | 5.75 | 0.65-50.60 | *0.115* |
| Yes vs no | 3 vs 136° | 6.10 | 0.75-49.58 | *0.091* |  |  |  |  |
| **Type** | 3 vs 103* | 7.20 | 0.86-59.96 | *0.068* |  |  |  |  |
| NMM vs SSM, LMM, Other | 4 vs 135° | 5.57 | 0.68-45.33 | *0.108* |  |  |  |  |
| **Mitotic count** | 29 vs 77* | 3.76 | 0.64-16.82 | *0.083* |  |  |  |  |
| >=1/mm^2 vs^ <1/mm^2^ | 34 vs 105° | 3.23 | 0.81-12.93 | *0..097* |  |  |  |  |
| **Ki67 positivity** | 13 vs 80* | 4.10 | 0.68-24.55 | *0.122* |  |  |  |  |
| >25% vs <=25% | 15 vs 107° | 3.55 | 0.65-19.37 | *0.143* |  |  |  |  |
| **KIRREL high vs low** | 27 vs 79 | 7.58 | 1.47-39.13 | ***0.016*** | 27 vs 79 | 6.32 | 1.19-33.65 | ***0.031*** |

*Only cases with evaluable KIRREL expression included in the analysis.

° Entire cohort of thin melanoma, including cases with evaluable KIRREL expression and cases which non-evaluable KIRREL expression.

Only variables with a p-value <=0.05 in the univariable analysis, in either the KIRREL-evaluated or in the extended cohort, were included in the multivariable analysis.

SSM = Superficial spreading melanoma, NMM = Nodular malignant melanoma, LMM = Lentigo malignant melanoma.
