# Supplementary figures and images for: Discovery of KIRREL as a biomarker for prognostic stratification of patients with thin melanoma
Source: Biomark Res. 2019 Jan 14;7:1. doi: 10.1186/s40364-018-0153-8 (PMC6332842; doi:10.1186/s40364-018-0153-8)

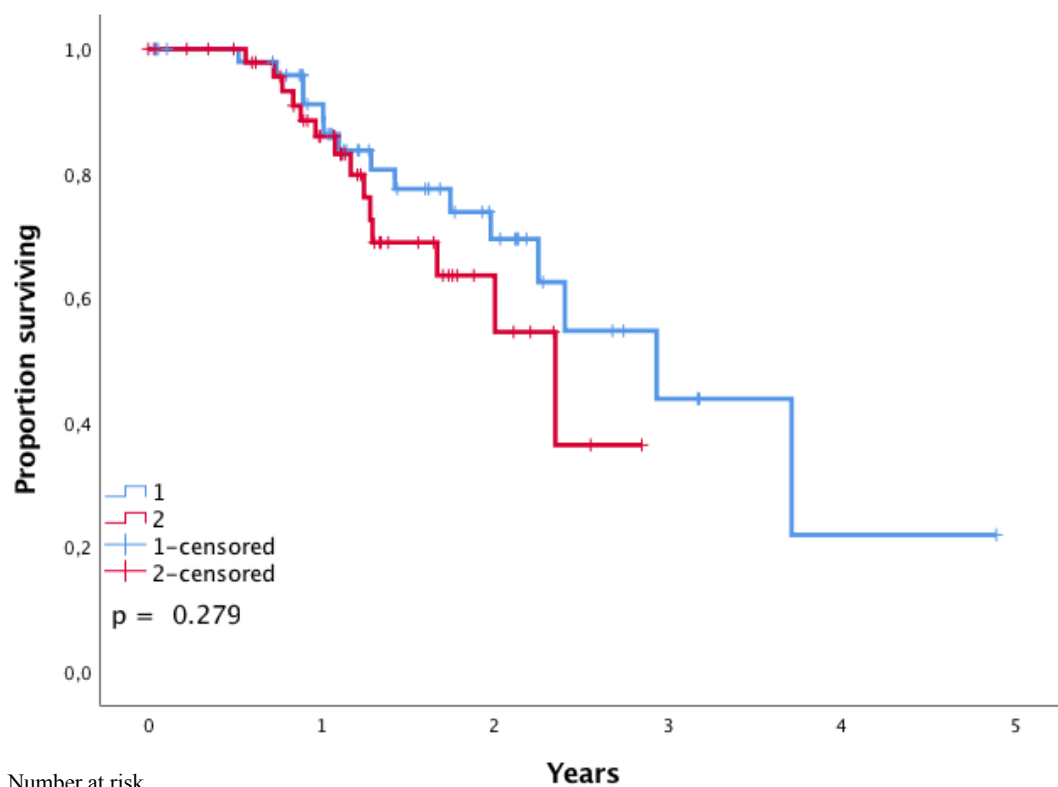

Supplement: Supplementary file 4 — Kaplan-Meier curve illustrating overall survival according to high and low mRNA expression (median cutoff) in TCGA dataset. (PDF 45 kb) [file 40364_2018_153_MOESM4_ESM.pdf]
